# Diaphragm Disease: The Limitation of Laparoscopy and Assessment of the Small Bowel for Strictures Using a Ball Bearing

**DOI:** 10.1100/tsw.2006.216

**Published:** 2006-09-07

**Authors:** C.E. Moffat, M.N. Khyan, C.G. Davies, A.S.K. Ghauri, C.J. Ranaboldo

**Affiliations:** Department of General Surgery, Salisbury District Hospital, Wiltshire SP2 8BJ, United Kingdom

**Keywords:** acute abdomen, diaphragm disease, diagnostic techniques, digestive system, emergencies, enteritis, ileum – pathology, ileal disease (chemically induced), pathology, inflammatory bowel disease – chemically induced, intestinal mucosa – pathology, intestinal obstruction – chemically induced, diagnosis, pathology, aetiology, surgery, laparoscopy – methods, utilization, laparotomy, minimally invasive surgical procedures, small intestine – pathology, surgery, drug effects, surgery

## Abstract

Diaphragm disease is a rare cause of intestinal obstruction that will be seen with increasing frequency with the widespread use of nonsteroidal anti-inflammatory drugs (NSAIDs). We present a case study of a patient with diaphragm disease where the diagnosis was not apparent at laparoscopy, and passage of a steel ball through the small intestine was required to identify all strictures present. A high index of suspicion, recognition of the limitations of conventional diagnostic aids, and the need to assess the full length of the small bowel are all important in the surgical management of this condition.

## INTRODUCTION

This case study reviews the management of a patient with multiple small bowel strictures secondary to chronic nonsteroidal anti-inflammatory drug (NSAID) use whose bowel had a normal appearance at laparoscopy. Laparotomy and the passage of a 15-mm diameter steel ball throughout the length of the small bowel were needed to identify all the areas of stricturing. With the widespread use of NSAIDs, this problem is likely be encountered more frequently.

## CASE REPORT

A 54-year-old woman presented as an emergency with abdominal pain and a 9-month history of cramping abdominal pain after meals, associated with weight loss. She had recently been investigated for a microcytic anaemia with a normal upper gastrointestinal endoscopy. She had been on regular diclofenac for 2 years for musculoskeletal pain, but was otherwise well.

On admission, she was systemically well with a soft, undistended abdomen and mild tenderness in her epigastrium. Routine blood tests were normal, and an abdominal ultrasound scan only demonstrated a distended thin-walled gall bladder with no stones.

After failure of initial conservative treatment, a CT scan was requested. This showed a small bowel obstruction with loops of oedematous, dilated small bowel adjacent to normal terminal ileum (Fig. 1), suggesting a possible adhesion at the level of the pelvic brim.

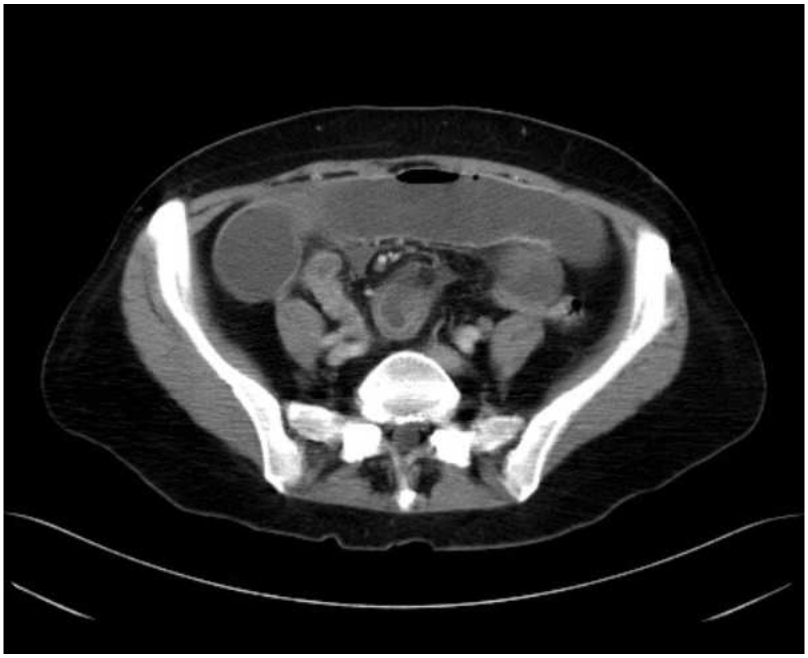

**FIGURE 1**

A diagnostic laparoscopy was performed, but at operation, no adhesions were found and her small bowel was examined in its entirety and appeared normal, with no change in calibre. No further procedure was undertaken.

After the laparoscopy, her symptoms continued and a gastrograffin meal was organised. This showed delayed passage of contrast with evidence of a partial distal ileal obstruction. Her symptoms persisted despite conservative treatment, and a formal barium meal and small bowel follow-through showed some resolution of the proximal small bowel dilatation seen previously, but a constant, mobile narrowing of the distal ileum was seen. This was thought to be consistent with a non malignant stricture with no adhesions.

A formal laparotomy was performed and although her small bowel appeared macroscopically normal, multiple small bowel strictures were evident on palpation near the ileocaecal junction (Fig. 2). A segment of bowel containing four strictures was resected (Fig. 3). Histology of the bowel subsequently confirmed the diagnosis of diaphragm disease.

Although her abdominal pain improved, she continued to complain of pain and occasional vomiting after eating, and underwent a further laparotomy 6 weeks later. An enterotomy was made at the site of a suspected stricture (Fig. 4) and a 15-mm steel ball bearing was passed through the length of her small bowel (Fig. 5). A further seven strictures were found. Four of these strictures were resected in a single segment of small bowel, and the remaining three underwent strictureplasty. Interestingly, not all of these strictures were palpable, and were only identified with the ball bearing. After the second laparotomy, her pain resolved and she was able to re-establish enteral intake and began to gain weight.

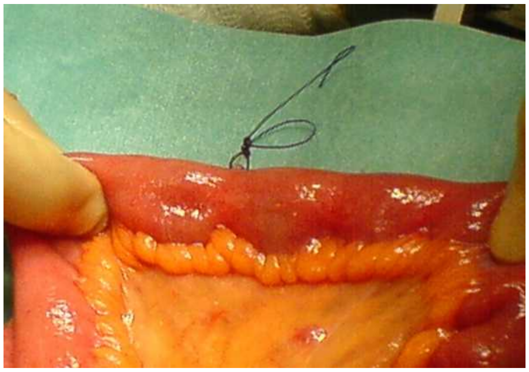

**FIGURE 2**

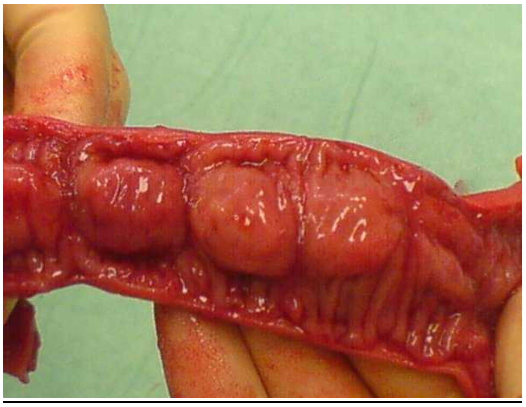

**FIGURE 3**

## DISCUSSION

Most clinicians are familiar with effects of NSAIDs on the gastroduodenal epithelium, but there is increasing evidence describing their effects on the distal intestine[1]. The spectrum of pathological changes includes stricture formation, ulceration, perforation, and haemorrhage[2]. Patients are usually asymptomatic, but can present with anorexia and weight loss, anaemia, or intestinal obstruction or perforation[3]. The term "diaphragm disease" is used for the presence of pathognomonic histopathological changes resulting in mucosal projections into the lumen with submucosal fibrosis[4]. It has been found that 70% of patients on long-term NSAIDs have evidence of small intestinal inflammation[5] but the actual incidence of diaphragm disease is still unknown[6].

The diagnosis of strictures is often protracted and 90% of the time made at laparotomy[7]. Diagnostic tests of small intestinal permeability, inflammation, or protein loss have been described to infer the presence of NSAID enteropathy in the laboratory[1,7], but they are not used routinely in clinical practice. Enteroscopy is time consuming, expensive, and provides poor anatomical localisation of diseased segments[1]. Capsule endoscopy carries the risk of precipitating bowel obstruction[8]. Contrast studies are limited by the inability to differentiate between strictures and exaggerated mucosal folds[7]. Even at laparotomy, the bowel may appear to be macroscopically normal as the serosal surface and mesentry is often unaffected[6] unlike in Crohns disease[7], and the strictures may be impalpable[9]. Identification of all strictures at laparotomy has been advocated either with intraoperative enteroscopy or the passage of a Foley catheter balloon[6], or by inflating the small bowel with carbon dioxide through a nasogastric tube allowing the strictured areas to become prominent[9].

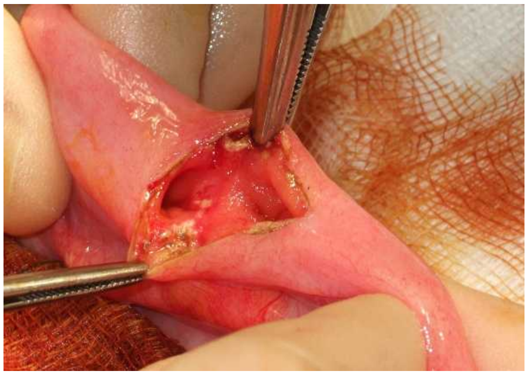

**FIGURE 4**

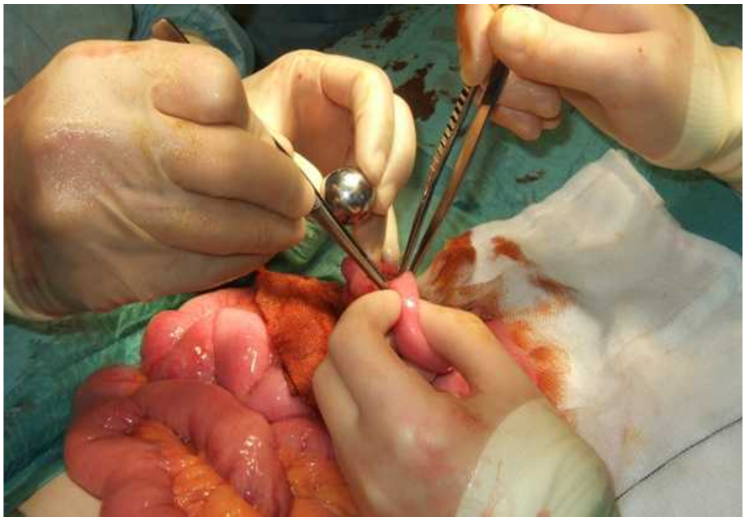

**FIGURE 5**

Treatment of strictures should include surgery (strictureplasty or resection) and withdrawal of the offending NSAID. However, continued symptoms from the mucosal inflammation can be seen to persist for up to 16 months after this[9]. There is some evidence that sulfasalazine, metronidazole, and misoprostil may be effective in reducing ongoing damage[10].

A high index of suspicion is required to prevent delay in making the diagnosis and for effective use of investigations in these patients. Although laparoscopy has a role in the management of patients with small bowel obstruction[11], in patients with suspected NSAID enteropathy, its role appears to be limited. At laparotomy, some strictures may be palpable, and it is then imperative that the entire bowel is assessed intraluminally to determine the presence of further strictures that may not be apparent.

Although the necessity of intraluminal examination of the bowel is more important than the specific technique used, we recommend the use of the ball bearing in assessing the small bowel as a simple, easy, low-risk technique, similar to the method described in the assessment of Crohn's strictures[12]. It does not require the expense or expertise of enteroscopy, and does not carry the risk of barotrauma related to insufflation of the bowel. In comparison to a Foley catheter balloon, it can be removed, reinserted, and passed remote from the original insertion point allowing treatment of strictures as they are discovered. It should be noted that it is important to place a soft bowel clamp at the proximal jejunum and distal ileum to prevent loss of the ball bearing into the duodenum or colon.

## CONCLUSION

Diaphragm disease is an important cause of small bowel obstruction in patients taking long-term NSAIDs. A high index of suspicion is needed to identify patients with this condition. Radiological studies may be misleading and not identify the complete range of strictures. Strictures may not be visible on inspection of the surface of the bowel and, therefore, laparoscopy is limited in its usefulness. At laparotomy, palpation of the bowel is inadequate in localising all strictures, and intraluminal assessment of the bowel is important. We recommend the use of a steel ball passed along the length of the small bowel to ensure adequate identification of strictures.
